# Cerebellar interpositus nucleus exhibits time-dependent errors and predictive responses

**DOI:** 10.1038/s41539-024-00224-y

**Published:** 2024-02-26

**Authors:** Gloria G. Parras, José M. Delgado-García, Juan Carlos López-Ramos, Agnès Gruart, Rocío Leal-Campanario

**Affiliations:** Division of Neurosciences, Universidad Pablo de Olavide, Seville, Spain

**Keywords:** Classical conditioning, Excitability, Perception

## Abstract

Learning is a functional state of the brain that should be understood as a continuous process, rather than being restricted to the very moment of its acquisition, storage, or retrieval. The cerebellum operates by comparing predicted states with actual states, learning from errors, and updating its internal representation to minimize errors. In this regard, we studied cerebellar interpositus nucleus (IPn) functional capabilities by recording its unitary activity in behaving rabbits during an associative learning task: the classical conditioning of eyelid responses. We recorded IPn neurons in rabbits during classical eyeblink conditioning using a delay paradigm. We found that IPn neurons reduce error signals across conditioning sessions, simultaneously increasing and transmitting spikes before the onset of the unconditioned stimulus. Thus, IPn neurons generate predictions that optimize in time and shape the conditioned eyeblink response. Our results are consistent with the idea that the cerebellum works under Bayesian rules updating the weights using the previous history.

## Introduction

Learning is a functional state of the brain that should be understood as a continuous process, rather than being restricted to the very moment of its acquisition, storage, or retrieval^1,2^. Learning is critical for survival, and requires the participation of numerous neural structures, as well as their proper and timed activation, including ultrastructural changes in synaptic strength. In this regard, the predictive coding framework assumes the brain works as a Bayesian inference system^3–5^ organized hierarchically, where higher stations are constantly trying to anticipate the future by generating predictions (likelihood-based in the priors), to minimize prediction errors^6^. Thus, from this framework learning process works by weakening the responses that encode prediction error.

The delayed classical conditioning of the eyelid responses is a well-known associative learning task that helps to generate a new motor ability^7–9^. In this form of associative learning, a neutral stimulus (conditioned stimulus, CS) overlaps and co-terminates with an eyeblink-elicited stimulus (unconditioned stimulus, US), resulting—over time—in the emergence of a conditioned eyelid response to the CS. This delayed conditioning paradigm allows prediction of the timing of contingent sensory events and is probably one of the most-investigated examples of the predictive learning process^10–12^.

Classical eyeblink conditioning depends on several cortical regions, including the hippocampus^13,14^, motor cortex^15–17^, and prefrontal cortex^18,19^. Some subcortical structures such as the claustrum^20^, the amygdala^21^, and the red nucleus^22–24^ are also involved in eyeblink conditioning. However, the cerebellum has also been classically considered a very important structure involved in the acquisition, storage, timing, and/or performance of conditioned eyelid responses^1,25,26^. The peculiar internal organization of the cerebellum and its connectivity with the cerebral cortex and brainstem nuclei is hypothesized to work as a predictive system^27,28^. This predictive system operates by comparing predicted states with actual states, learning from errors, and updating its internal representations to generate predictions that minimize future errors^12^. In particular, a discrepancy between the expected outcome and the actual outcome may result in prolonged changes in neuronal activity patterns, referred as long-lasting error responses. This extended response, persisting beyond the immediate occurrence of an error, contributes to the brain’s optimization of future responses. Specially, the IPn of the cerebellum receives inputs from the inferior olive, pontine nuclei, and Purkinje cells, which supposedly relay information about the functional characteristics of the stimuli used as US and CS, and modify the cerebellar output sent to various areas of the brain, such as the red nucleus and thalamus, which control the motor response associated with the CS^23,29,30^. Nevertheless, most of the works addressing the implication of active inference in the cerebellum are based on time windows restricted to CS-US presentations^10,25,31^, and the emergence of predictive responses, and prediction errors, at the IPn, have not yet been addressed.

In the present work, we used a delay conditioning paradigm, but considering the time both before and after the CS-US, as an additional approach to decipher the role of the IPn in the generation of neuronal predictive responses and error suppression. We studied the neuronal and the orbicularis oculi EMG activity at three specific conditioning sessions, named initial -first conditioning session-, halfway -fifth conditioning session- and final -tenth and last conditioning session. Our results demonstrate that IPn neurons exhibit a long-lasting error response for almost one second after the CS-US offset when learning is not established. These error responses were significantly reduced in the middle (halfway) stage of the learning process, while the predictive response of the US occurred once the learning is well established. A neuronal long-lasting error response refers to sustained neuronal activity resulting from an error in the processing of information, which needs to be minimized. These results prove that the IPn works as an active inference system linking action and perception. Hence, the present study underscores the dynamic nature of learning and provides novel insight into the neuronal mechanisms underlying the acquisition and refinement of new motor abilities.

## Results

**Fig. 1:**
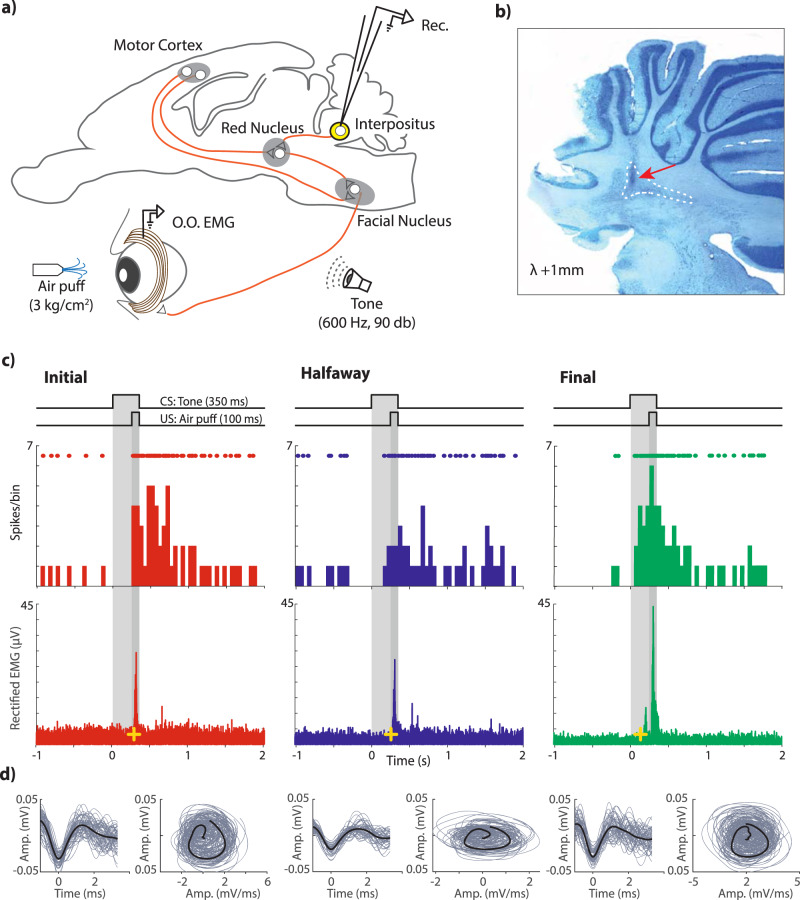
Experimental design. **a** Schematic diagram of the rabbit brain and eye illustrating the recording sites. **b** A representative photomicrography of a Nissl-stained brain section illustrating the track left by a glass micropipette. The dotted line outlines IPn boundaries. **c** Representative examples of three putative IPn neurons and their corresponding O.O. EMG for successive conditioning sessions. Red, blue, and green data correspond to the initial, halfway, and final conditioning sessions, respectively. From top to bottom are illustrated the CS (tone), the US (air puff), the firing activity of the selected neurons, their firing rates (spikes/bin), and the O.O. EMG. Yellow crosses indicate the precise moment at which a significant voltage change was detected in the recorded EMG (see “Methods”): initial = 276.8 ms; halfway = 263.2 ms, and final = 141.6 ms latencies with relation to CS presentation. **d** Spike waveform (left) and phase-space portrait (right) for each of the illustrated neurons. Spike sorting is based on a 24 D-vector for the first derivative in the time domain and phase-space^32^.

A total of 188 (initial = 62, halfway = 60, and final = 66) single units from awake rabbits were recorded and analyzed. Recording electrodes were aimed at the left IPn, in an area from the posterior part of the anterior interpositus to the rostral part of the posterior one (Fig. 1a, b). Recorded units were selected and identified following spike sorting procedures described in Methods (Fig. 1d) and already published in a previous work^32^. Although earlier descriptions in mice^33^ and cats^34^ have reported the presence of IPn neurons inhibited during CS-US presentations in conditioned animals, the number of inhibitory neurons recorded in the present work was rather small. In accordance, we decided to concentrate our analysis on activated units (Fig. 1c). The percentage of conditioned responses was measured as the occurrence of an eyelid response preceding the US presentation, but at least 50 ms following the CS presentation. As previously reported^16,35^ we found an increase in conditioned responses across the successive conditioning sessions, with a percentage of conditioned responses for the initial (non-conditioned stage), halfway, and final (conditioned stage) sessions reported as 7%, 25%, and 81%, respectively.

### IPn neurons change their firing patterns during the acquisition process

**Fig. 2:**
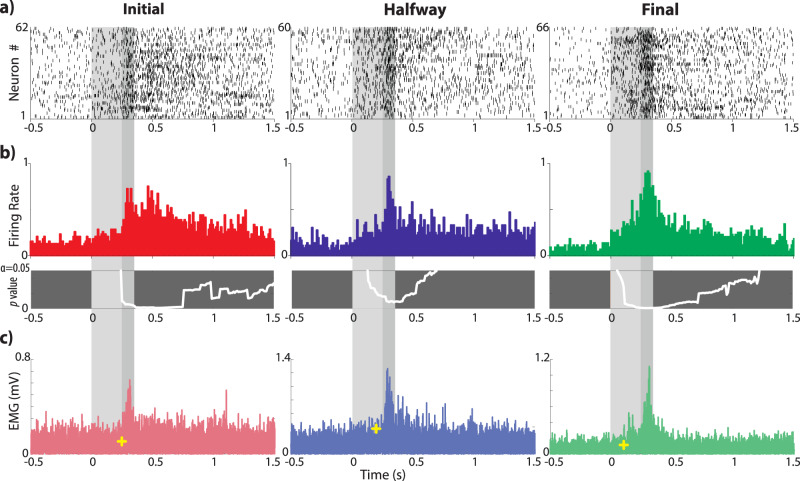
Neuronal and rectified EMG responses across classical eyeblink conditioning sessions. **a** Dot rasters of the recorded single IPn neurons during initial, halfway, and final conditioning sessions. Each dot on a row represents an action potential generated by one neuron during the corresponding trial. The Y-axis indicates the total number of neurons plotted and included in the analysis. Shadowed areas indicate the timing of CS (light gray) and CS-US (dark gray) presentations. **b** PSTH distribution of neuronal spike times for the three recorded conditioning sessions (initial in red, halfway in blue, and final in green; bin size 10 ms). The bottom panels show the instantaneous *p* value (white trace) of the corresponding PSTH (Wilcoxon signed-rank test for 300 comparisons, corrected for FDR = 0.1 and smoothed. The critical threshold for significance was set at *p* = 0.05 and is represented as the upper boundary of the box). **c** Rectified and averaged O.O. EMG. The yellow crosses indicate the precise moment at which we found an abrupt change in the voltage of the EEG signal. Note that significant EMG changes happened at 245.2 ms, 199.0 ms, and 102.8 ms after tone onset for non-conditioned, halfway, and conditioned sessions, respectively. Thus, animals learned to anticipate the eyeblink to avoid the US acting on the cornea. We found analogous decreases in the latency of IPn neuron activation with respect to CS presentation.

To examine the pattern response of IPn neurons during the acquisition process, (i.e., in the initial, middle, and final conditioning sessions), we computed a dot raster of the spike trains exhibited by each recorded neuron (Fig. 2a). Visual inspection revealed that, during the initial session, IPn neurons agglutinate their spikes around the US, and for a long time after the end of the US. However, this pattern change in the final session, with neuron firing linked to the moment before and during the US.

To analyze this changing pattern and its relation to eyelid movement, the peri-stimulus time histogram (PSTH) and the analysis over time of each corresponding session were computed (Fig. 2b). Lower panels in Fig. 2b show the instantaneous *p*-value (white trace) of the corresponding neuronal spike count (Wilcoxon signed-rank for 300 comparisons, FDR-corrected) revealing the moments where IPn neurons responded significantly differently from zero. Results demonstrate that IPn neurons remained significantly active for a long time after the end of the US across all conditioning sessions. Moreover, the first time-bin with a significant *p* value occurs at 250 ms (*p* = 0.0094), 130 ms (*p* = 0.0358), and 60 ms (*p* = 0.0491) after the CS onset for initial, halfway, and final conditioning sessions respectively. Thus, IPn neurons started to predict the occurrence of a US during the halfway conditioning session. However, this predictive response is well established in the final conditioning where IPn neurons respond well in advance of the US.

We also computed the averaged-rectified *Orbicularis Oculi* (O.O.) EMG of each corresponding session and calculated the precise moment at which a significant change occurs in the EMG (Fig. 2c). Results show that the EMG activity is brought forward by conditioning. Yellow crosses indicate a significant start at 254.2 ms, 199 ms, and 102,8 ms after CS onset for the initial, halfway, and final conditioning sessions, respectively. That is, the eyelid movement starts ≈ 150 ms in advance once the conditioning is well established. After matching data illustrated in Fig. 2b, c, it appears that the firing rate of IPN neurons starts before the detected significant changes in the O.O. EMG (yellow cross in Fig. 2c) by 4.2 ms for the initial, 69 ms for halfway, and 42.8 ms for final conditioning sessions.

This is the first time that a long period after and before the delay paradigm has been studied. The aim was to better understand the timing responses covered by IPn neurons. For this, we divided the recorded spike trains (Fig. 2a, b) into four successive time windows: (i) *spontaneous*, from -1 s to 1 ms preceding tone onset; (ii) CS: *tone*, from tone onset to 250 ms after; (iii) CS-US: *tone-air puff*, from 251 ms to 350 ms; and (iv) *late* period, from 351 ms to 2 s after the end of the US.

### The IPn increases predictions and reduces prediction-errors across learning

From the predictive coding framework, the firing activities of IPn neurons could be seen as part of the brain’s predictive coding mechanism, which adjusts its predictions based on the expected outcome of the stimulus. Thus, changes in the discharge rate through conditioning sessions are relevant because their presence posits that the IPn could generate predictions about incoming sensory information and use them to minimize prediction errors.

**Fig. 3:**
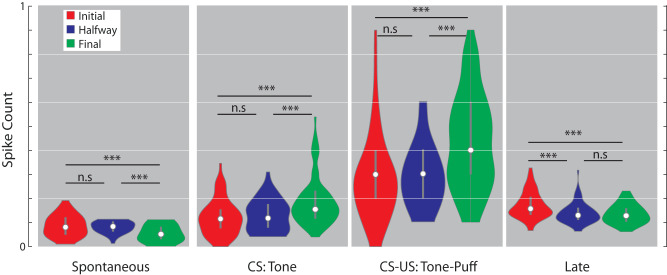
Violin plots representing the binned spike count for each time window (spontaneous, CS, CS-US, and late) and conditioning session (initial, halfway, and final). The white dot and the gray line inside each distribution indicate the median and the interquartile range. Asterisks denote statistically significant differences between conditioning sessions (n.s., non-significant, *p < 0.05, **p < 0.01, ***p < 0.001).

Then, to determine whether neurons in the IPn change their firing rate across learning, we calculated the average spike count and the ranksum test per time windows (in 10 ms bins) and conditioning sessions (Fig. 3). Our findings reveal that during the spontaneous time window, there was a decrease in the spike count as conditioning progresses (mean for initial = 0.0872, halfway = 0.07786, and final = 0.0533). The *p* values for comparison between initial-halfway, halfway-final, and initial-final were 0.4543, 3.21^−5^, and 1.82^−5^, respectively. However, we observed an increase in spike count from halfway through the final conditioning session for both the CS and CS-US time windows. The mean for CS was 0.1203 for initial, 0.1276 for halfway, and 0.1964 for late. The *p* values for initial-halfway, halfway-final, and initial-final were 0.4879, 2.19^−4^, and 2.14^−5^, respectively. The mean for CS-US was 0.2855 for initial, 0.29 for halfway, and 0.427 for late. The *p*-values for initial-halfway, halfway-final, and initial-final were 0.5964, 6.7^−5^, and 2.92^−5^, respectively.

Finally, we observed a reduction in spike count from the beginning (initial) to the middle (halfway) of the conditioning session in the late time window. The mean for the late time window was 0.1717 for initial, 0.1368 for halfway, and 0.1335 for late. The *p* values for initial-halfway, halfway-final, and initial-final were 6.33^−5^, 0.8775, and 1.06^−4^, respectively.

Those results indicate that neurons in the IPn adjust their responses by reducing the firing in the moments prior (spontaneous) and post (late) to the stimulus presentation, and by increasing the firing rate during the CS-US presentation across the learning process. Thereby, the IPn reduces the error responses and generates predictive responses during the present associative learning task.

### IPn neurons optimize the spiking activity throughout the learning process

**Fig. 4:**
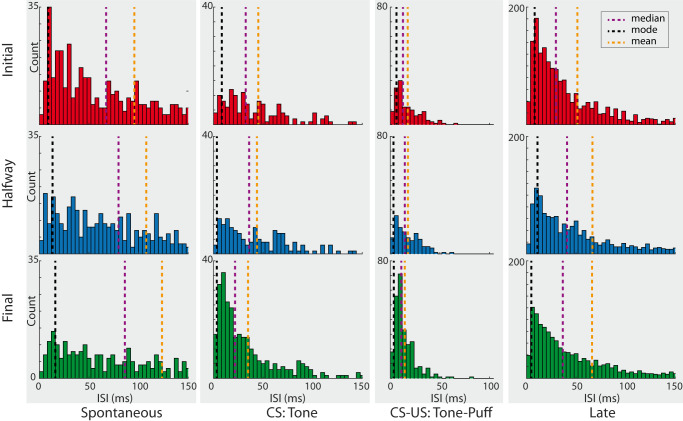
ISI histograms for each time window and conditioning session. The first row is for the initial, the second row for halfway, and the third row for final conditioning sessions. Dotted lines indicate the mode, median, and mean of the ISIs distribution. Note that ISIs become longer at spontaneous and late windows across conditioning, while ISIs become shorter during CS presentations. These results indicate that the IPn optimizes its firing activity to predict the US.

To evaluate the history-dependency of the spikes, we performed the same time-windowing procedure for the ISI. Figure 4 presents the ISI histograms for each of the four previously described time windows (with a bin size of 4 ms), each conditioning session. Our analysis revealed that most of the ISIs were consistently shorter during the CS, CS-US, and late time windows by approximately 5 ms (mode for initial sessions: CS = 8.4 ms, CS-US = 6.2 ms; halfway sessions: CS = 3.4 ms, CS-US = 3.2; and final sessions: CS = 3.3 ms; CS-US = 3.5 ms). However, in the spontaneous window, the ISIs became longer (mode for initial sessions = 8.9 ms; halfway sessions = 13.2 ms; and final sessions = 16 ms). Interestingly, ISIs during the tone-air puff time window showed no significant changes. These findings suggest that IPn neurons optimize their spiking activity and respond predictably across learning.

In general, the relationship between the findings presented in the previous text and predictive coding theory highlights the IPn capacity to optimize its responses to sensory information through learning processes, ultimately reducing the prediction error, and improving its ability to process and interpret sensory information.

### IPn neurons refine their spiking activity across learning

**Fig. 5:**
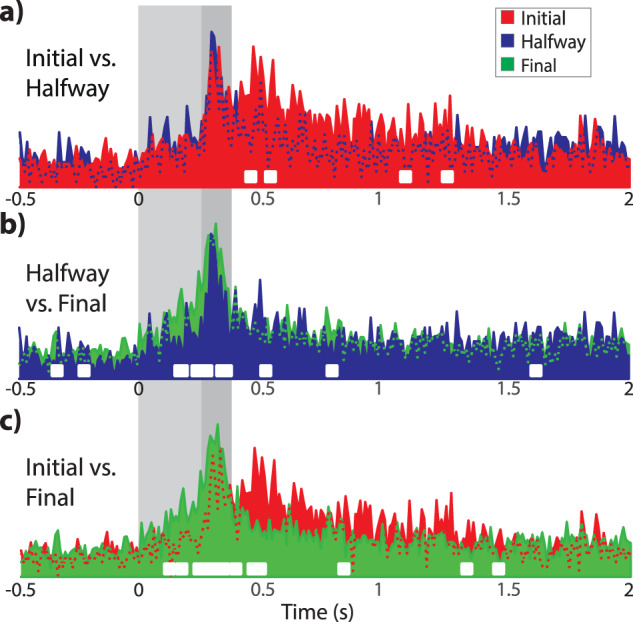
Comparisons between the firing patterns of IPn neurons across the successive conditioning sessions. **a** Averaged binned spike count profiles for initial vs. halfway conditioning sessions (same data as in Fig. 2). White squares indicate the precise moment when the comparison between the two conditioning sessions is significantly different (two-sample *t* test, *p* < 0.01). **b**, **c** Same as in (**a**) but computed for halfway vs. final, and for initial vs. final, respectively. Note that from initial to halfway there was a significant reduction in firing rates following the end of the US (**b**), while the emergence of the conditioned response occurred once learning was well established (**c**).

Furthermore, to better understand the overall dynamic response of IPn neurons, we performed a bin-by-bin (in 10 ms bin size) comparison between the averaged histograms alongside the full recording (Fig. 5). Comparisons between the initial and halfway conditioning sessions (Fig. 5a) show significant differences (white squares) only after the CS-US presentation finished (*p* < 0.01). In contrast, in Fig. 5b, c, halfway vs. final, and initial vs. final conditioning sessions, we found significant differences mainly during stimulus presentation.

Overall this result demonstrates that IPn neurons shape their firing activity across conditioning sessions. Moreover, neuronal conditioned responses emerge once the learning is well established. As a result, the responses in the late period typical of the initial phase of the learning process disappear and are agglutinated around the stimulus presentation during the middle and final phase of the learning.

### Training improves the synchrony between IPn and eyelid closure

Despite the aforementioned results, there is still much debate around the role of the cerebellum in this type of learning. Some authors argue the cerebellum is the place where the eyelid-conditioned response is generated and stored, while others maintain that the cerebellum is just part of the many brain structures contributing to the generation and storage of conditioned eyelid responses^8^.

**Fig. 6:**
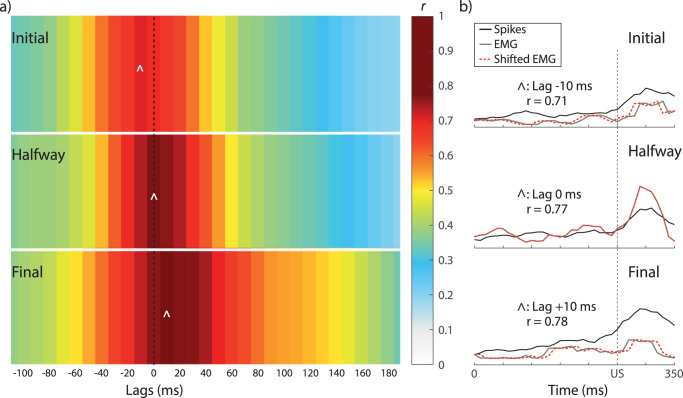
Cross-correlation analysis between the firing rate of IPn neurons and the O.O. EMG across conditioning sessions. **a** A colormap illustrating the correlation coefficient magnitude corresponding to time lags between –100 ms and 180 ms. The rectified O.O. EMG was displaced in steps of 10 ms with respect to the neuronal firing rate. In agreement with this, each block represents a 10 ms lag. Data are shown from top to bottom for the initial, halfway, and final sessions. **b** A representation of the highest cross-correlation index indicated in (**a**) with caret symbols (^). Black and gray lines represent the averaged spike count and the rectified EMG during the stimulus representation, respectively. From top to bottom, the highest *r* coefficient value occurs when the EMG is shifted backward –10 ms (for initial), 0 ms (for halfway), and +20 ms (for final), respectively. Note that the highest *r* values move from negative to positive lags across conditioning. Similarly, the number of lags with *r* values > 0.6 increases with the conditioning process. These results indicate that the IPn tunes a temporary transformation during the acquisition of a newly learned motor response.

Therefore, to determine whether the IPn is (or not) the main generator of conditioned eyelid responses, we computed the cross-correlation between the averaged rectified EMG and the averaged spike count for the 350 ms of CS-US presentation (Fig. 6). If maximum correlations are found at positive lags, then we could deduce that the O.O. EMG precedes IPn neural activities; the opposite case would indicate that changes in the firing rate of IPn neurons lead O.O. EMG muscle activation to generate the conditioned response.

As illustrated in Fig. 6a, we found that the majority of stronger correlations occurred with negative lags during the initial conditioning session, while in the final conditioning, the higher correlations are predominantly positive. Analogously, we also found a larger number of lags with correlation indexes >0.6 (warmer colors) during the final sessions. In Fig. 6b we represent the averaged spike count (black line), the averaged-rectified O.O. EMG (gray line), and the shifted O.O. EMG (red dotted line) that correspond to the highest correlation coefficient, shown in Fig. 2a with a caret symbol (^). The strongest correlation in the initial conditioning session (*r* = 0.7063) is found when the EMG has shifted backward 10 ms. In contrast, during the halfway session, the maximum coefficient was observed at lag 0 (*r* = 0.7746)—that is, during the middle process of the learning, the two signals were strongly related. Importantly, in the final session, maximum cross-correlation was found at lag +1 (*r* = 0.7781). Taken together, these results indicate that classical conditioning modifies and improves the synchrony between IPn neuronal activities and the O.O EMG during the acquisition of the conditioned response. That is, the IPn nucleus is involved in the precise performance of conditioned eyelid responses once the learning is well established.

### IPn neurons works as a Bayesian system

Finally, to conclusively assess the capacity of IPn single neurons to exhibit predictive activity, we analyzed trials in which the US is omitted, termed as CS-alone trials. If the IPn activity is solely caused by the US, the response to CS-alone should remain consistent across conditioning sessions (initial, halfway, and final). Conversely, under the predictive coding framework, we should observe a significant response to CS-alone, meaning that IPn works as a Bayesian inference system^36^, minimizing errors and enhancing predictive capabilities.

**Fig. 7:**
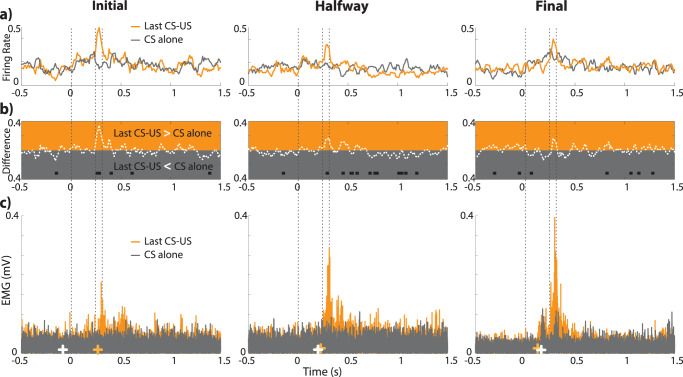
Comparisons between last paired CS-US trials and CS alone trials across conditioning sessions. **a** Neuronal responses in the PSTH profile distribution for the last paired CS-US trials (orange) and CS alone trials (gray). **b** Firing rate differences between the last CS-US and CS alone sessions, with black squares indicating statistically significant moments (*p* < 0.05, Wilcoxon signed rank test). **c** Averaged and rectified O.O. EMG for the last paired CS-US trials (orange) and the CS alone (gray). Crosses denote precise moments of signal changes (white for the CS alone and orange for the CS-US). Note that statistically significant differences in the period where the US occurs (or is omitted), vanishes across the successive conditioning sessions. These results indicate that, when animals are well conditioned, there are not differences at the neuronal level between the presence or the omission of the US. Furthermore, the EMG activity delineates the progression of the conditioned eyelid responses.

To address this question, we computed the averaged binned firing rate for the CS-alone trials and the last CS-US trial before the CS-alone, as well as the difference between them, for all three conditioning sessions— initial, halfway and final (Fig. 7a, b). To ensure an equal number of trials contributed to the resulting difference, only the last CS-US trial before the CS-alone was considered. Results indicate a significant difference during the initial session, specifically at 260–280 ms, corresponding to the moment in which the US -air puff- occurs. Similarly, in the halfway conditioning session, a significant difference is found at 290 ms. Conversely, during the final session, there are no differences at the time when the US occurs. However, a significantly higher response is found 60 ms after the CS onset (black squares in Fig. 7b).

This data is reinforced by the analysis of eyelid responses (EMG activity). We found not only eyelid conditioned responses when the last CS-US occurs – as described previously for the entire population in Fig. 2c—but also that the generation of a genuine eyelid conditioned response during the CS-alone at the final conditioning session. Yellow and white crosses (CS-US and CS-alone, respectively) indicate the precise moment in which the O.O. EMG is detected (from initial to final sessions, EMG for the last CS-US trials is significant at 267 ms, 208 ms, and 129 ms; while for the CS-alone, the EMG is significant at –80 ms, 200 ms, and 149 ms, respectively from initial to final sessions).

Overall, the present study provides novel insights into the changes in the activity of IPn neurons during the acquisition process of conditioned eyelid responses. The reported findings suggest that the changes in the IPn firing pattern, reducing errors and increasing predictive responses, are fundamental to properly generating the conditioned response. Furthermore, it highlights the importance of considering the timing of neuronal firing in relation to the acquired conditioned eyelid responses.

## Discussion

**Fig. 8:**
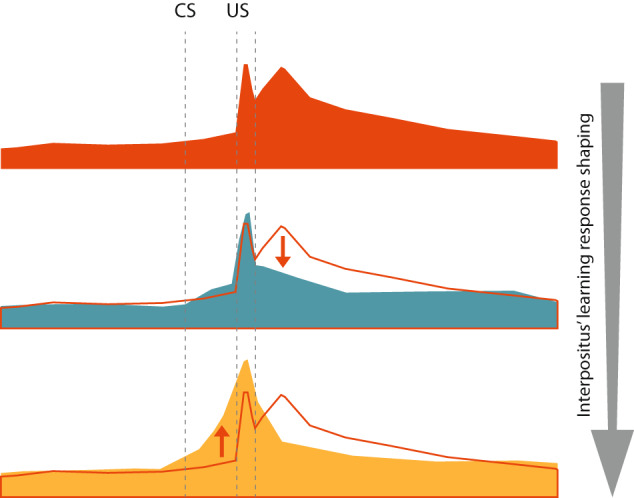
A diagrammatic representation of the modulation of IPn neuronal responses across learning. From top to bottom are shown the temporal time-courses of IPn neuronal responses across initial (orange), halfway (cyan), and final (yellow) conditioning sessions. The orange outline is the silhouette of the initial conditioning session. The illustrated profiles are cartoon representations from the actual averaged data. Note the IPn activity is sustained in time after the stimulus offset before learning acquisition. Afterward, this sustained late response is reduced, and finally, the conditioned response is generated.

This study demonstrates that IPn neurons exhibit a long-lasting error response time after the end of CS-US presentation, during the initial conditioning session—namely before the acquisition of the conditioned response. These error responses are significantly reduced in the middle (halfway session) stage of learning; afterward, IPn neurons generate predictive (i.e., conditioned) responses of US presentation during the final conditioning session, when learning is well established. Thus, the IPn firing pattern is learning-stage-dependent. Furthermore, our data demonstrate that temporal integration and associative encoding are evident in the firing pattern of IPn single neurons. Consequently, IPn neurons respond to the behavioral relevance of the stimuli, using past experience to optimize the firing pattern (Fig. 8).

In accordance with the foregoing, the firing pattern of IPn neurons matches the Bayesian inference, where the response to current stimuli is based on the likelihood of the previous experience; thereby allowing the generation of a prediction of the upcoming US. Moreover, the learning-related reduction of the long-lasting error response observed after the CS-US can be considered similar to the conditioned diminution described in other works restrictive to the US^37^. However, for a correct inference, a hierarchical organization is fundamental^38^. In this regard, cerebellum connectivity with many other brain areas is precisely followed. It is generally accepted that climbing fibers send up the information about the US to the Purkinje cells and the IPn, while pontine nuclei send the CS information through mossy fibers to granular cells and parallel fibers in the cerebellar cortex and the IPn^7,9,23,39–41^. Thus, the CS and US converge at the cerebellar cortex and IPn. However, the IPn is the sole output of the cerebellum that drives the newly learned response^10,42–44^.

Because mossy fibers and climbing fibers directly or indirectly excite the IPn, our results agree with works that suggest error signals arrive from the inferior olive through the climbing fibers, and that those error signals are ignored when a conditioned response is established^45^. The reinforcement of the inhibitory feedback between the IPn and the inferior olive through conditioning sessions will refine the firing response^28^.

Hence, the acquisition of a new motor response would be initially refined by the double inhibitory pathway between the inferior olive, Purkinje cells, and IPn neurons. This is supported by experiments that demonstrate IPn activation exerts a powerful inhibition of the inferior olive and that all olivary neurons undergo a long-lasting silence^33,46–48^. Besides, the number of excitatory synapses between the mossy fibers and IPn neurons increases with delay eyeblink conditioning sessions^49–52^ which enhances IPn neurons’ ability to activate the red nucleus.

The ability of IPn neurons to shape their firing around the stimulus after training indicates that IPn neurons are context-dependent. This outcome can be formulated as prediction-error adjustment in the context of active inference^36^. Such assumption is supported by studies using trace eyeblink conditioning, where the conditioned response depends on the length of the interval between the offset of the CS and the onset of the US^53^, validating that the CS under the delay paradigm propitiates an effective context to predict an outcome.

Furthermore, previous studies on the firing activities of rostral medial prefrontal (mPFC) neurons reported an increase in the firing rate in middle conditioning sessions^18^. This functional finding, together with the reported direct projections from mPFC to pontine nuclei^54,55^, suggests that the IPn receives a downstream influence from upper cortical stations to update the current model. Thus, under the predictive coding framework, we can assume that in middle sessions we observe the optimization of neuronal and neuromuscular activity to suppress errors based on top-down generative models; whereas in the final conditioning session what we observe is the optimization of synaptic gain and efficacy to encode the predictions and cause the conditioned response.

Importantly, many structures have been reported to play a role in associative learning—some of them related to attention and cognitive aspects^16,19,35,56^. Altogether, the evidence suggests a hierarchical connectivity between the cerebellum and forebrain areas that is fundamental for correct associative learning.

Our experimental design uses an auditory stimulus as CS. This is relevant because it has been demonstrated that auditory information is hierarchically processed independently of the awareness state^57^. Furthermore, auditory studies report that PFC responsiveness is driven by unpredictability, and its response is delayed, exhibiting long-lasting prediction-error-signaling^58^. Therefore, it would be reasonable to consider that the firing patterns observed in IPn neurons during this associative learning task using an auditory stimulus as CS, align with previous studies supporting the hierarchical organization of the entire system involved in the acquisition of classical eyelid conditioning^7–9,25,59–61^. This assumption is reinforced by the cancelation of the conditioning eyeblink after the inhibition of the rostral mPFC^18^.

Interestingly, the firing profiles of IPn neurons reported here presented their best correlations with a delay of 20 ms during the final session—i.e., when the conditioned response was already present. Similar results were found in other species^10,31^, which suggests that IPn neurons are not completely leading, but optimize conditioned eyelid responses. In the same regard, it has been reported that pyramidal cells of the motor cortex of classically conditioned rabbits fire up to 110 ms in advance to the beginning of conditioned eyeblinks^16^, while—as reported here—IPn neurons preceded conditioning responses only to a maximum of 40 ms. In accordance, the role of IPn in classical eyeblink conditioning should be mainly related to the proper performance and/or reinforcement of conditioned responses, but not to their initiation^7,8,34^.

This study provides the first direct evidence of sensory prediction errors in the IPn, which play a role in updating the internal model to learn and predict unexpected sensory outcomes. Although recent studies suggest that cerebellum-dependent learning and timing are more complex than previously thought^27^, advancing beyond the basic understanding of eyeblink classical conditioning presents a significant overarching challenge for the field.

## Methods

### Experimental subjects

Experiments were performed on four adult male rabbits (New Zealand white albino) provided by an authorized supplier (Isoquimen, Barcelona, Spain). The animals were 2.5–3 months old and weighed ≈ 2.5 kg on their arrival at the Pablo de Olavide Animal House. Following their arrival, animals were housed individually until the end of the experiments. They were kept on a 12-h light/dark cycle with constant ambient temperature (21 ± 0.5 °C) and humidity (55 ± 5%) and had food and water *ad libitum*.

### Ethics statement

Experimental procedures were approved conforming to the standards of the Animal Care Committee of the Pablo de Olavide University (reference 06/03/2018/025), the European Directive 2010/63/EU, the Spanish Royal Decree 53/2013 (BOE 34:11370-421, 2013), and the ARRIVE guidelines for the use of laboratory animals in behavioral experiments.

### Surgery

We induced and maintained anesthesia with a ketamine-xylazine mixture (25 mg/kg Ketamidor + 3 mg/kg Xylasol for i.m. induction; and 10 mg/kg Ketamidor + 3 mg/kg Xylasol in 100 mL of Lactato-RingerVet for i.v. maintenance at a flow rate of 10 mg/kg/h). We also applied atropine sulfate (0.5 mg/kg, s.c.) to reduce bronchial secretions.

Animals were implanted with two Teflon-coated stainless steel (50 µm Ø) hook electrodes in the upper eyelid to record the electromyographic (EMG) activity of the orbicularis oculi (O.O.) muscle. Then, we performed a craniotomy ( ≈ 4 × 4 mm) in the left parietal bone to expose the area overlying the IP, taking as reference coordinates 0.5 mm anterior and 5 mm lateral from lambda^62,63^. The dura was removed, and an acrylic recording chamber was constructed around the opened window (Fig. 1a).

### Recordings

We recorded simultaneously the O.O. EMG and the firing activities of IPn neurons. Recording sessions began two weeks after surgery. Each rabbit was habituated to stay in a Perspex restrainer box specially designed to limit the animal’s movements. The box was placed on the recording set-up, which was located in a softly illuminated and noise-attenuated room.

The EMG activity of the O.O. muscle was recorded using a Grass P511 amplifier (Grass-Telefactor) with a bandwidth of 0.1–10 kHz, sampled at 5 kHz. Neuronal activity was recorded utilizing borosilicate glass capillaries with filament (Harvard Apparatus model EC1 30-0083; manufactured tip length 2.5 cm and 2 µm Ø) and filled with 2 Μ NaCl (2–4 MΩ). The neural signal was sampled at 25 kHz, and filtered 1–10 kHz. The depth from the brain surface to record IPn neurons was between 16 and 18 mm from lambda. At the end of each recording session, the glass micropipette was removed, and the cerebellar surface was cleaned and protected with bone wax and a plastic cover. Consequently, the spikes reported in the present study do not originate from the same neuron recorded across different sessions.

### Classical conditioning

Classical conditioning was achieved using a delay paradigm. The conditioned stimulus (CS) was a tone (350 ms, 600 Hz, and 90 dB), and the unconditioned stimulus (US) was an air puff (100 ms, 3 kg/cm^2^) starting 250 ms after CS onset (Fig. 1a, c). All animals received a total of 10 conditioning sessions, one per day. Each conditioning session consisted of 60 CS-US trials and 6 CS alone. Hence, there were a total of 66 trials with an interval between trials of 60 s (±5 s). Consequently, during each conditioning session, there was a 90.9% probability of a CS being followed by a US, while the probability of a CS-alone occurrence was 9.1%. This design allows the establishment of a regularity, occasionally disrupted by the omission of the US, in a deterministic manner.

We aimed to determine the involvement of IPn neurons across the evolution of classical conditioning learning. Since we wanted to minimize experimental disturbance of the acquisition process, we recorded neuronal activity and O.O. EMG only from alternative sessions (2- initial, 5- halfway, and 10- final) to avoid excessive damage to the IPn.

### Histology

At the end of the experiments, animals were deeply anesthetized with sodium pentobarbital (50 mg/kg, i.p.) and perfused transcardially with saline and paraformaldehyde (0.2 M, pH 7.4). Brains were removed and processed for Nissl staining in slices of 50 µm to identify the recording sites. The proper location of EMG electrodes was also checked (Fig. 1b).

### Analysis

The un-rectified EMG activity of the O.O. muscle, the unitary activity of IPn neurons, and 1-volt rectangular pulses corresponding to CS and US presentations were acquired online through an analog-to-digital converter (CED 1401-plus, CED, Cambridge, UK), and transferred to a computer for quantitative offline analysis. Neuronal data were acquired using the Spike2^©^ program (from CED) and divided into three-second frames using Signal^©^ (also from CED). Frames without significant responses were excluded from further analysis. Then, neuronal activity from subsequent frames was clustered by spike sorting with the VISSOR^™^ software. The spike sorting was based on the shape, phase, and distribution of each recorded spike as previously described by some of us^32^. The ensuing clustering is considered the putative IPn neurons reported in this work. Forward analysis for recorded neurons, as well as for EMG activity, was computed using Matlab^™^ software with built-in functions, the Statistics and Machine Learning toolbox, or custom scripts developed in our laboratory.

The averaged O.O. EMG was high-pass filtered (100 Hz) and DC was removed and the signal rectified using the Matlab ‘detrend’ and ‘abs’ functions, respectively. To determine the precise moment at which an abrupt change in the EMG signal took place we used a ‘findchangepts’ function, creating two regions that minimize the sum of the residual (squared) error of each region from its local mean.

Since we look primarily for enhancement of responses to stimulus presentation, we consider here only activated neurons. A peri-stimulus time histogram of neuronal activity was computed for each recording session (initial, halfway, and final) from 1 s before to 2 s after tone onset (Fig. 2a, b and Fig. 7a). A Wilcoxon signed-rank test was performed to check whether each data point was different from zero. The corresponding *p*-values were corrected for false discovery rate (FDR = 0.01) using the Benjamini-Hockberg test for multiple comparisons. The corrected *p*-values were then smoothed (‘smooth’ function in Matlab at 0.8 spans) for visual improvement of the multitude of data points (lower panel in Fig. 2b). Later, the averaged PSTH was divided into four time windows (Figs. 3, 4) to find out the precise timing contribution of IPn neurons to the generation of conditioned eyelid responses: (i) the *spontaneous* window that comprises events occurring before the CS onset and ranges between –1 s and –1 ms; (ii) the *tone* window, which comprises events ranging between 0 and 250 ms, is the timing between the tone onset and just before the US onset; (iii) the *tone-air puff* window, comprising events where the tone (CS) and the air puff (US) match in time, ranging from 251 ms to 350 ms; and (iv) the *late* window, which includes events between 351 ms and 2 s. A Wilcoxon ranksum test was used to analyze differences between time windows.

Correlation coefficients of EMG and spikes were computed using the ‘corrcoef’ function of Matlab and then fitting a linear regression. Averaged spike count comparisons between sessions were performed using the ‘*ttest2’* function of Matlab and the Wilcoxon ranksum test. A bin-by-bin comparison for conditioning sessions was performed for neural and EMG activity to determine whether there were significant differences between the sessions for neuronal activity and the EMG responses.

To conclusively identify neuronal error responses and the presumed error minimization across the learning process, we compared the average firing rates of CS-alone trials (Initial *n* = 14, Halfway *n* = 23, and Final *n* = 16) with the last paired CS-US trials before the CS-alone (Initial *n* = 14, Halfway *n* = 27, and Final *n* = 20) (Fig. 7). This procedure ensures probabilistic fairness. The difference between both averaged neuronal firing rates (last paired CS-US minus CS-alone) was calculated using the ‘minus’ function in MATLAB (Fig. 7b). Then two-sample *t* test (‘’ttest2’ in MATLAB) was performed to identify statistically significant differences in each 10 ms time bin (time bines within *p* < 0.05 are indicated by black squares in Fig. 7b) for each conditioning session. The O.O. EMG of the corresponding trials was computed as described above (Fig. 7c).

### Reporting summary

Further information on research design is available in the Nature Research Reporting Summary linked to this article.

### Supplementary information

Reporting summary

## Data Availability

The data that support the findings of this study are available from the corresponding author upon reasonable request.

## References

[1] Delgado-García JM, Gruart A (2002). The role of interpositus nucleus in eyelid conditioned responses. Cerebellum.

[2] Delgado-García JM, Gruart A (2017). Learning as a functional state of the brain: Studies in wild-type and transgenic animals. Adv. Exp. Med. Biol..

[3] Parr T, Friston KJ (2018). The anatomy of inference: Generative models and brain structure. Front. Comput. Neurosci..

[4] Friston K (2005). A theory of cortical responses. Philos. Trans. R. Soc. B Biol. Sci..

[5] Clark A (2013). Whatever next? Predictive brains, situated agents, and the future of cognitive science. Behav. Brain Sci..

[6] Hohwy, J. *The Predictive Mind*. (Oxford University Press, 2013). 10.1093/acprof:oso/9780199682737.001.0001.

[7] Manto M (2012). Consensus paper: Roles of the cerebellum in motor control-the diversity of ideas on cerebellar involvement in movement. Cerebellum.

[8] Parras GG, Leal-Campanario R, López-Ramos JC, Gruart A, Delgado-García JM (2022). Functional properties of eyelid conditioned responses and involved brain centers. Front. Behav. Neurosci..

[9] De Zeeuw CI, Ten Brinke MM (2015). Motor learning and the cerebellum. Cold Spring Harb. Perspect. Biol..

[10] Sánchez-Campusano R, Gruart A, Delgado-García JM (2009). Dynamic associations in the cerebellar–motoneuron network during motor learning. J. Neurosci..

[11] Koekkoek SKE (2003). Cerebellar LTD and learning-dependent timing of conditioned eyelid responses. Sci. (80-.).

[12] Gandolfi D (2022). Emergence of associative learning in a neuromorphic inference network. J. Neural Eng..

[13] Múnera A, Gruart A, Muñ Oz MD, Fernández-Mas R, Delgado-GARCÍA JM (2001). Hippocampal pyramidal cell activity encodes conditioned stimulus predictive value during classical conditioning in alert cats. J. Neurophysiol..

[14] McEchron MD, Disterhoft JF (1997). Sequence of single neuron changes in CA1 hippocampus of rabbits during acquisition of trace eyeblink conditioned responses. J. Neurophysiol..

[15] López-Ramos JC, Delgado-García JM (2021). Role of the motor cortex in the generation of classically conditioned eyelid and vibrissae responses. Sci. Rep..

[16] Ammann C, Márquez-Ruiz J, Gómez-Climent M, Delgado-García JM, Gruart A (2016). The motor cortex is involved in the generation of classically conditioned eyelid responses in behaving rabbits. J. Neurosci..

[17] Hasan MT (2013). Role of motor cortex NMDA receptors in learning-dependent synaptic plasticity of behaving mice. Nat. Commun..

[18] Leal-Campanario R, Delgado-García JM, Gruart A (2013). The rostral medial prefrontal cortex regulates the expression of conditioned eyelid responses in behaving rabbits. J. Neurosci..

[19] Weiss C, Disterhoft JF (2011). Exploring prefrontal cortical memory mechanisms with eyeblink conditioning. Behav. Neurosci..

[20] Reus-García MM (2021). The Claustrum is Involved in Cognitive Processes Related to the Classical Conditioning of Eyelid Responses in Behaving Rabbits. Cereb. Cortex.

[21] Burhans LB, Schreurs BG (2008). Inactivation of the central nucleus of the amygdala abolishes conditioning-specific reflex modification of the rabbit (oryctolagus cuniculus) nictitating membrane response and delays classical conditioning. Behav. Neurosci..

[22] Pacheco-Calderón R, Carretero-Guillén A, Delgado-García JM, Gruart A (2012). Red nucleus neurons actively contribute to the acquisition of classically conditioned eyelid responses in rabbits. J. Neurosci..

[23] Freeman JH, Steinmetz AB (2011). Neural circuitry and plasticity mechanisms underlying delay eyeblink conditioning. Learn. Mem..

[24] Desmond JE, Moore JW (1991). Single-unit activity in red nucleus during the classically conditioned rabbit nictitating membrane response. Neurosci. Res..

[25] Takehara-Nishiuchi K (2018). The anatomy and physiology of eyeblink classical conditioning. Curr. Top. Behav. Neurosci..

[26] Christian KM, Thompson RF (2003). Neural substrates of eyeblink conditioning: acquisition and retention. Learn. Mem..

[27] Hull C (2020). Prediction signals in the cerebellum: Beyond supervised motor learning. eLife.

[28] Ito M (2013). Error detection and representation in the olivo-cerebellar system. Front. Neural Circuits.

[29] Gonzalez-Joekes J, Schreurs BG (2012). Anatomical characterization of a rabbit cerebellar eyeblink premotor pathway using pseudorabies and Identification of a local modulatory network in anterior interpositus. J. Neurosci..

[30] Morcuende S, Delgado-García JM, Ugolini G (2002). Neuronal premotor networks involved in eyelid responses: retrograde transneuronal tracing with rabies virus from the orbicularis oculi muscle in the rat. J. Neurosci..

[31] López-Ramos JC, Houdek Z, Cendelín J, Vožeh F, Delgado-Garciá JM (2018). Timing correlations between cerebellar interpositus neuronal firing and classically conditioned eyelid responses in wild-type and Lurcher mice. Sci. Rep..

[32] Caro-Martín CR, Delgado-García JM, Gruart A, Sánchez-Campusano R (2018). Spike sorting based on shape, phase, and distribution features, and K-TOPS clustering with validity and error indices. Sci. Rep..

[33] ten Brinke MM (2017). Dynamic modulation of activity in cerebellar nuclei neurons during pavlovian eyeblink conditioning in mice. Elife.

[34] Gruart A, Guillazo-Blanch G, Fernández-Mas R, Jiménez-Díaz L, Delgado-García JM (2000). Cerebellar posterior interpositus nucleus as an enhancer of classically conditioned eyelid responses in alert cats. J. Neurophysiol..

[35] Leal-Campanario R, Fairén A, Delgado-García JM, Gruart A (2007). Electrical stimulation of the rostral medial prefrontal cortex in rabbits inhibits the expression of conditioned eyelid responses but not their acquisition. Proc. Natl. Acad. Sci. USA.

[36] Friston K, Kiebel S (2009). Predictive coding under the free-energy principle. Philos. Trans. R. Soc. B Biol. Sci..

[37] Goodman AM, Harnett NG, Knight DC (2018). Pavlovian conditioned diminution of the neurobehavioral response to threat. Neurosci. Biobehav. Rev..

[38] Friston K (2011). What is optimal about motor control?. Neuron.

[39] De Zeeuw CI, Lisberger SG, Raymond JL (2021). Diversity and dynamism in the cerebellum. Nat. Neurosci..

[40] Kelly RM, Strick PL (2003). Cerebellar loops with motor cortex and prefrontal cortex of a nonhuman primate. J. Neurosci..

[41] Perciavalle V (2013). Consensus paper: Current views on the role of cerebellar interpositus nucleus in movement control and emotion. Cerebellum.

[42] McCormick DA, Thompson RF (1984). Neuronal responses of the rabbit cerebellum during acquisition and performance of a classically conditioned nictitating membrane-eyelid response. J. Neurosci..

[43] Mauk MD, Steinmetz JE, Thompson RF (1986). Classical conditioning using stimulation of the inferior olive as the unconditioned stimulus. Proc. Natl. Acad. Sci. USA.

[44] Hesslow G, Svensson P, Ivarsson M (1999). Learned movements elicited by direct stimulation of cerebellar mossy fiber afferents. Neuron.

[45] Ohmae S, Medina JF (2015). Climbing fibers encode a temporal-difference prediction error during cerebellar learning in mice. Nat. Neurosci..

[46] Bazzigaluppi P, Ruigrok T, Saisan P, de Zeeuw CI, de Jeu M (2012). Properties of the nucleo-olivary pathway: An in vivo whole-cell patch clamp study. PLoS One.

[47] De Zeeuw CI (1998). Microcircuitry and function of the inferior olive. Trends Neurosci.

[48] ten Brinke MM, Boele HJ, De Zeeuw CI (2019). Conditioned climbing fiber responses in cerebellar cortex and nuclei. Neurosci. Lett..

[49] Ohyama T, Nores WL, Medina JF, Riusech FA, Mauk MD (2006). Learning-induced plasticity in deep cerebellar nucleus. J. Neurosci..

[50] Boele HJ, Koekkoek SKE, De Zeeuw CI, Ruigrok TJH (2013). Axonal sprouting and formation of terminals in the adult cerebellum during associative motor learning. J. Neurosci..

[51] Kleim JA (2002). Synapse formation is associated with memory storage in the cerebellum. Proc. Natl. Acad. Sci. USA.

[52] Weeks ACW (2007). Eye-blink conditioning is associated with changes in synaptic ultrastructure in the rabbit interpositus nuclei. Learn. Mem..

[53] Clark RE, Martin SJ (2018). Current topics in behavioral neurosciences 37 behavioral neuroscience of learning and memory. Curr. Top. Behav. Neurosci..

[54] Buchanan SL, Thompson RH, Maxwell BL, Powell DA (1994). Efferent connections of the medial prefrontal cortex in the rabbit. Exp. Brain Res..

[55] Schmahmann JD, Pandya DN (1997). Anatomic organization of the basilar pontine projections from prefrontal cortices in rhesus monkey. J. Neurosci..

[56] Fiocchi FR, Dijkhuizen S, Koekkoek SKE, De Zeeuw CI, Boele HJ (2022). Stimulus Generalization in Mice during Pavlovian Eyeblink Conditioning. eNeuro 9, ENEURO.

[57] Parras GG (2017). Neurons along the auditory pathway exhibit a hierarchical organization of prediction error. Nat. Commun..

[58] Casado-Román L, Carbajal GV, Pérez-González D, Malmierca MS (2020). Prediction error signaling explains neuronal mismatch responses in the medial prefrontal cortex. PLOS Biol.

[59] Thompson RF, Thompson JK, Kim JJ, Krupa DJ, Shinkman PG (1998). The nature of reinforcement in cerebellar learning. Neurobiol. Learn. Mem..

[60] Cheron G, Márquez-Ruiz J, Dan B (2016). Oscillations, timing, plasticity, and learning in the cerebellum. Cerebellum.

[61] Bracha V (2009). The cerebellum and eye-blink conditioning: Learning versus network performance hypotheses. Neuroscience.

[62] Shek, J. W., Wen, G. Y. & Wisniewski, H. M. Atlas of the Rabbit Brain and Spinal Cord. (Basel: Karger, 1986). 10.1159/isbn.978-3-318-05386-9.

[63] Girgis, M. & Wang, S.-C. A new stereotaxic atlas of the rabbit brain. 70 (W.H. Green, 1981).

