# Supplementary material for: Cerebellar interpositus nucleus exhibits time-dependent errors and predictive responses
Source: NPJ Sci Learn. 2024 Feb 26;9:12. doi: 10.1038/s41539-024-00224-y (PMC10897197; doi:10.1038/s41539-024-00224-y)
Supplement: Supplementary file 1 — Reporting summary [file 41539_2024_224_MOESM1_ESM.pdf]

nature portfolio

Corresponding author(s): Parras GG; and Leal-Campanario R.

Last updated by author(s): Jan 10, 2024

Reporting Summary

Nature Portfolio wishes to improve the reproducibility of the work that we publish. This form provides structure for consistency and transparency in reporting. For further information on Nature Portfolio policies, see our Editorial Policies and the Editorial Policy Checklist.

Statistics

For all statistical analyses, confirm that the following items are present in the figure legend, table legend, main text, or Methods section.

n/a	Confirmed	
☐	☒	The exact sample size (*n*) for each experimental group/condition, given as a discrete number and unit of measurement
☒	☐	A statement on whether measurements were taken from distinct samples or whether the same sample was measured repeatedly
☐	☒	The statistical test(s) used AND whether they are one- or two-sided<br>*Only common tests should be described solely by name; describe more complex techniques in the Methods section.*
☒	☐	A description of all covariates tested
☐	☒	A description of any assumptions or corrections, such as tests of normality and adjustment for multiple comparisons
☐	☒	A full description of the statistical parameters including central tendency (e.g. means) or other basic estimates (e.g. regression coefficient) AND variation (e.g. standard deviation) or associated estimates of uncertainty (e.g. confidence intervals)
☐	☒	For null hypothesis testing, the test statistic (e.g. F, t, r) with confidence intervals, effect sizes, degrees of freedom and P value noted<br>*Give P values as exact values whenever suitable.*
☐	☒	For Bayesian analysis, information on the choice of priors and Markov chain Monte Carlo settings
☒	☐	For hierarchical and complex designs, identification of the appropriate level for tests and full reporting of outcomes
☐	☒	Estimates of effect sizes (e.g. Cohen's *d*, Pearson's *r*), indicating how they were calculated

Our web collection on statistics for biologists contains articles on many of the points above.

Software and code

Policy information about availability of computer code

Data collection	Spike2.
Data analysis	Signal Analysis; Matlab and VISSOR.

For manuscripts utilizing custom algorithms or software that are central to the research but not yet described in published literature, software must be made available to editors and reviewers. We strongly encourage code deposition in a community repository (e.g. GitHub). See the Nature Portfolio guidelines for submitting code & software for further information.

Data

Policy information about availability of data

All manuscripts must include a data availability statement. This statement should provide the following information, where applicable:

- Accession codes, unique identifiers, or web links for publicly available datasets
- A description of any restrictions on data availability
- For clinical datasets or third party data, please ensure that the statement adheres to our policy

The data that support the findings of this study are available from the corresponding author upon reasonable request.

Research involving human participants, their data, or biological material

Policy information about studies with human participants or human data. See also policy information about sex, gender (identity/presentation), and sexual orientation and race, ethnicity and racism.

Reporting on sex and gender	n/a
Reporting on race, ethnicity, or other socially relevant groupings	n/a
Population characteristics	n/a
Recruitment	n/a
Ethics oversight	n/a

Note that full information on the approval of the study protocol must also be provided in the manuscript.

Field-specific reporting

Please select the one below that is the best fit for your research. If you are not sure, read the appropriate sections before making your selection.

☒ Life sciences ☐ Behavioural & social sciences ☐ Ecological, evolutionary & environmental sciences

For a reference copy of the document with all sections, see nature.com/documents/nr-reporting-summary-flat.pdf

Life sciences study design

All studies must disclose on these points even when the disclosure is negative.

Sample size	We statistically determined the sample size of neurons to obtain a statistical power of 0.8 using a 'sampsizepwr' function in Matlab.
Data exclusions	No data were excluded from the present study. However, during the recording and spike sorting, only those excitatory neurons within a refractory period higher than 3.33 ms were considered to ensure that no artifacts were computed.
Replication	Different animals, and the effect of sample size, are enought to assure replication.
Randomization	The neuronal collection was completely randomized during each recording session when the pipette was introduced.
Blinding	To avoid bias, we present both raw data and processed data over an extended time period. The data were reviewed by all the authors.

Reporting for specific materials, systems and methods

We require information from authors about some types of materials, experimental systems and methods used in many studies. Here, indicate whether each material, system or method listed is relevant to your study. If you are not sure if a list item applies to your research, read the appropriate section before selecting a response.

Materials & experimental systems

n/a	Involved in the study	
☒	☐	Antibodies
☒	☐	Eukaryotic cell lines
☒	☐	Palaeontology and archaeology
☐	☒	Animals and other organisms
☒	☐	Clinical data
☒	☐	Dual use research of concern
☒	☐	Plants

Methods

n/a	Involved in the study	
☒	☐	ChIP-seq
☒	☐	Flow cytometry
☒	☐	MRI-based neuroimaging

Animals and other research organisms

Policy information about studies involving animals; ARRIVE guidelines recommended for reporting animal research, and Sex and Gender in Research

Laboratory animals	adult male rabbits (New Zealand white albino) provided by an authorized supplier (Isoquimen, Barcelona, Spain). The animals were 2.5-3 months old and weighed [illegible] 2.5 kg on their arrival at the Pablo de Olavide Animal House.
Wild animals	n/a
Reporting on sex	n/a
Field-collected samples	Following their arrival, animals were housed individually until the end of the experiments. They were kept on a 12-h light/dark cycle with constant ambient temperature (21 ± 0.5 °C) and humidity (55 ± 5%) and had food and water ad libitum.
Ethics oversight	Experimental procedures were approved conforming to the standards of the Animal Care Committee of the Pablo de Olavide University (reference 06/03/2018/025), the European Directive 2010/63/EU, the Spanish Royal Decree 53/2013 (BOE 34:11370-421, 2013), and the ARRIVE guidelines for the use of laboratory animals in behavioral experiments.

Note that full information on the approval of the study protocol must also be provided in the manuscript.

Plants

Seed stocks	n/a
Novel plant genotypes	n/a
Authentication	n/a
